# Applying Persuasive Design Techniques to Influence Data-Entry Behaviors in Primary Care: Repeated Measures Evaluation Using Statistical Process Control

**DOI:** 10.2196/humanfactors.9029

**Published:** 2018-10-11

**Authors:** Justin St-Maurice, Catherine Burns, Justin Wolting

**Affiliations:** 1 Systems Design Engineering University of Waterloo Waterloo, ON Canada; 2 Applied Health Information Science Program School of Health & Life Sciences and Community Services Conestoga College Institute of Technology and Advanced Learning Kitchener, ON Canada; 3 Guelph Family Health Team Guelph, ON Canada

**Keywords:** data collection, data entry, data accuracy, health care, persuasive design, persuasive systems design framework, user interface

## Abstract

**Background:**

Persuasive design is an approach that seeks to change the behaviors of users. In primary care, clinician behaviors and attitudes are important precursors to structured data entry, and there is an impact on overall data quality. We hypothesized that persuasive design changes data-entry behaviors in clinicians and thus improves data quality.

**Objective:**

The objective of this study was to use persuasive design principles to change clinician data-entry behaviors in a primary care environment and to increase data quality of data held in a family health team’s reporting system.

**Methods:**

We used the persuasive systems design framework to describe the persuasion context. Afterward, we designed and implemented new features into a summary screen that leveraged several persuasive design principles. We tested the influence of the new features by measuring its impact on 3 data quality measures (same-day entry, record completeness, and data validity). We also measured the impacts of the new features with a paired pre-post *t* test and generated XmR charts to contextualize the results. Survey responses were also collected from users.

**Results:**

A total of 53 users used the updated system that incorporated the new features over the course of 8 weeks. Based on a pre-post analysis, the new summary screen successfully encouraged users to enter more of their data on the same day as their encounter. On average, the percentage of same-day entries rose by 10.3% for each user (*P*<.001). During the first month of the postimplementation period, users compensated by sacrificing aspects of data completeness before returning to normal in the second month. Improvements to record validity were marginal over the study period (*P*=.05). Statistical process control techniques allowed us to study the XmR charts to contextualize our results and understand trends throughout the study period.

**Conclusions:**

By conducting a detailed systems analysis and introducing new persuasive design elements into a data-entry system, we demonstrated that it was possible to change data-entry behavior and influence data quality in a reporting system. The results show that using persuasive design concepts may be effective in influencing data-entry behaviors in clinicians. There may be opportunities to continue improving this approach, and further work is required to perfect and test additional designs. Persuasive design is a viable approach to encourage clinician user change and could support better data capture in the field of medical informatics.

## Introduction

### Background

Technology can be designed to trigger emotional responses in humans, which can lead them to interact with technology as if it were a social actor. Behavior change techniques, such as persuasive design, can be used to intentionally design technology to change people’s attitudes and behaviors by leveraging social processes [1,2].

There are many advantages to using technology to influence behavior change: technology can automatically deploy persuasion strategies in real-time as users are performing a task; technology is persistent and consistent; technology can be deployed anonymously; and technology can exist in locations and contexts that are not possible for humans. Persuasive design can also quickly adapt to large amounts of data, simultaneously attempt several modalities to influence people, and can quickly scale once successful [1-3]. To date, the use of persuasive design in health care has focused primarily on consumer-facing mobile apps and has aimed to improve health outcomes [4]. In contrast, there are few examples of using persuasive design to influence clinician behavior within clinical systems, such as within an electronic medical record system.

Despite an absence of studies exploring the use of persuasive design to change clinicians’ behaviors, several studies have shown that basic social processes, such as persuasion and social comparisons, can successfully initiate behavior change. For example, a successful approach called audit-based education consisted of group meetings and presenting comparative data to individual physicians [5] and was described as the most successful change agent for influencing clinicians’ attitudes and habits regarding data entry [6]. More recently, a data quality feedback tool generated comparative data quality metrics between practices; data recording behavior and data quality improved significantly through the use of social comparisons between users [7].

Since clinicians can be influenced to change data-entry practices through social comparisons, and since persuasive design is intended to allow technology to emulate and facilitate these types of social processes, we hypothesize that persuasive design is a suitable approach to motivate clinicians to enter higher quality data into electronic systems. Conceptually, persuasive design can be leveraged to change clinician attitudes and behaviors regarding data-entry tasks.

### Data Entry in Primary Care

Primary care is an important part of the health care ecosystem. Primary care data is unique because it includes a patient’s entire health history and may extend from the patient’s birth until their death. As such, primary care data can be used for secondary purposes such as auditing, quality improvement, health service planning, epidemiological study, research, and measuring care quality [8,9]. Primary care data has also been used in novel ways to investigate challenging and broad health system problems [10-13]. However, the effective secondary use of this data is contingent on its quality.

There are many barriers to entering high-quality structured data into an electronic medical record. These barriers include user skill gaps, task time, and professional and organizational priorities [6,14]. The crux of the challenge with data quality in primary care is that clinicians are often asked to structure their data by clinic managers and consultants, but prefer writing unstructured narratives [15]. Entering structured data is also challenged by a lack of perceived value for future uses by clinicians. In many cases, clinicians do not fully accept the merit of entering structured data [16], and this negatively impacts data quality for secondary uses. One important finding in the literature is that the completeness and accuracy of primary care data often rely on the enthusiasm of clinicians [17]. The user interface for structured data entry is often simple and can facilitate the creation of structured data with minimal training; entering usable data requires appropriate entry behaviors and attitudes of clinician users.

In a previous study [18], several data quality benchmarks were developed based on the historical analysis of entries in a system designed to measure the effectiveness and costs of services. The study found that while 97.4% of the entries were valid (ie, logically consistent), only 21.7% of the entries were considered complete (ie, users had entered all the necessary information). As well, only 50.7% of the entries had been recorded on the same day as the clinical encounter. The study also described corollaries between data validity, data completeness, and data timeliness and concluded that entries were more likely to be valid and complete if they had been entered on the same day as the clinical encounter.

As health care reforms aim to improve the efficiency of care, organizations need to find ways to track the effectiveness, quality, and cost of care and services. This data is critical and cannot be accurately captured through free text and unstructured narratives. Organizations must continue to ensure that clinical documentation exists to serve patients, and they must also find ways to capture high-quality data for secondary use. Information systems and human processes need to adapt to evolving requirements and data needs.

### Systems Analysis and Persuasive Design

Cognitive work analysis (CWA) is a systems analysis framework that facilitates the analysis of the environment at various levels of detail and assesses how the environment impacts and shapes the human-information interaction. CWA is a systematic method that can be used to examine work activities of participants in workflows and processes with environmental, organizational, and social lenses [19].

CWA is well suited to consider the sociotechnical relationships between information systems and human processes and is an effective tool for designing systems for changing environments. CWA is broken down into 5 stages of analysis: work domain analysis, control task analysis, strategies analysis, social organization and co-operation analysis, and work competencies analysis [19]. Each stage of CWA provides a different level of detail for a complete analysis of a domain.

Oinas-Kukkonen and Harjumaa developed the persuasive systems design framework [3,20] to facilitate the identification and incorporation of persuasion principles into effective designs. Persuasive system design uses the idea of a persuasion context to define *how* users could be persuaded. Persuasive system design does not, however, link directly to a specific systems analysis framework; a designer needs to identify *who* the users are and *why* the change is required before they can build an effective persuasion context.

Recently, efforts have been made to link CWA to the persuasive system design framework [21,22]. Since the CWA framework provides a systematic approach to understanding ecology and cognition, it easily addresses many of the information requirements described by Oinas-Kukkonen and Harjumaa [3,20]. As well, the idea of tying CWA to Fogg Behavior Model [23] and persuasive system design has previously been explored by Rezai and Burns [24], though with only a few phases of the CWA framework.

Importantly, current literature has recently started to draw a link between CWA and the persuasive system design framework, providing a set of tools covering the complete analysis-to-design spectrum of persuasive design.

### Study Objective

Previous studies have shown that entries are more valid and complete if they are entered on the same day. However, only 50.7% of entries were recorded on the same day [18,21]. Thus, there is a need to find ways to influence clinician’s behaviors around data entry and data quality. The required behavior changes include encouraging users to enter their data on the same day as their patient encounter, encouraging users to enter a complete entry within the structured form, and encouraging error-free entries. Our objective was to expose clinicians to persuasive design in order to modify their data-entry behaviors.

## Methods

### Study Design

During our study, we analyzed the persuasion context of a primary care data-entry task. Following the analysis, we designed and implemented an updated user interface that implemented new persuasive features. Finally, we tested the influence of the new user interface on clinicians’ data recording behaviors and its impact on data quality.

### Study Context

In Ontario, there are over 200 family health teams. These organizations are Ontario’s implementation of team-based care. Family health teams employ allied health professionals, such as nurses, dietitians, social workers, and pharmacists. Allied health professionals provide supplementary services (such as one-on-one counseling and group therapy classes) to patients in the community. Patients are referred to allied health professionals by their family doctor at no cost. Family health teams are intended to improve the quality of primary care services and access to primary care physicians.

Family health teams must report the activities of their allied health professionals to the Government as a condition of funding. Though some of this information is available within medical records, extracting the information in a format that aligns with the reporting requirements is challenging. As well, electronic medical records are not easily adapted to new reporting requirements. Furthermore, if organizations have more than a single electronic medical record for documentation purposes, the challenges associated with collecting consistent data is compounded. In this context, family health teams with numerous allied health professionals working in multiple locations have opted to create separate systems to collect data for reporting purposes. These types of systems require allied health professionals to answer short survey questions for each clinical encounter. The collected data is aggregated to generate reports for the government and internal process improvement.

**Figure 1:**
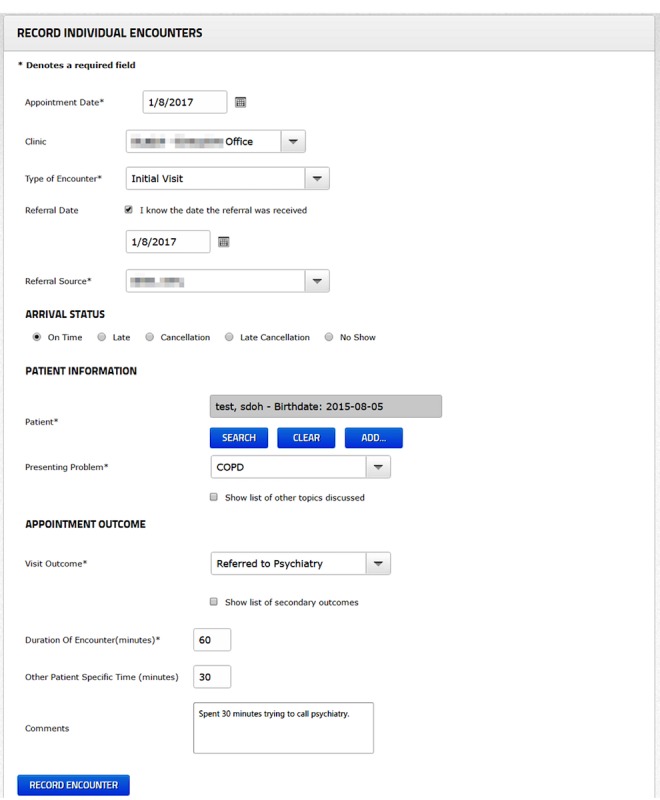
Screenshot of the reporting tool data entry screen.

One family health team (the “organization”) uses a separate data collection system (the “reporting system”) to collect data from its allied health professionals. The organization’s reporting system is an excellent example of a structured data-entry prompt, and it parallels the processes and use-cases of structured data entry in electronic medical records. Since the reporting system is incorporated into normal workflows, the tool is an interesting opportunity to explore data-entry behaviors in clinicians and benchmark data quality [21]. The organization’s current data-entry screen is shown in Figure 1.

The organization had a staff of approximately 110 and served 20 different family practices and 90 doctors in the community. A total of 53 employees were active users of the reporting system and used the system at least once per month. The organization was interested in finding ways to improve data quality in its reporting system. Based on a previous study, only 50.7% of the entries within the data collection system were recorded on the same day as the clinical encounter by allied health professionals [18]. As a primary goal, the organization wanted to introduce persuasive design to increase the number of entries that were entered on the same day as the clinical encounter. The organization saw improving the validity and completeness of the data in the reporting system as secondary objectives.

We worked with the organization to understand the sociotechnical context of the allied health professionals’ data recording task and developed a new user interface for the reporting tool to improve data quality. Over the course of several months, we measured the impact of the user interface changes.

### Analysis of Persuasion Context

To describe the persuasion context, we linked the CWA systems analysis framework to the persuasive system design design framework. We used data from a CWA conducted over the course of another study [21], where a CWA was completed regarding the reporting system’s data-entry tasks. Based on the results of the work domain analysis phase, we had access to several abstraction hierarchies that showed relationships between the organization’s goals, benchmarks, professional norms, and impacts on population health outcomes [14].

Based on the results of the control task analysis and strategies analysis phases, which described decision making regarding data entry and strategies employed by users to accomplish the work, we had decision ladders and information flow maps to describe user decision making and strategy adoption [14,21]. Each CWA model helped identify the user’s ecosystem and elements that would influence their behavior.

In persuasive system design, several principles are intended to support persuasive design. To identify which principles would be appropriate within the persuasion context, we used a CWA to inform a who, what, where, when, how (WWWWH) paradigm. Our ecological approach to persuasive design takes advantage of the strengths of each framework: CWA provides insight about *context, ecology, and cognition*; Fogg Behavior Model provides information about *when* the change will occur; and persuasive system design provides *tools and design* ideas that can create a change in behavior. The combination of these frameworks filled the analysis-to-design spectrum with a series of useful tools and sources of information. This was a novel approach to filling the analysis-to-design gap and generated a useful design concept to implement and test.

To link our models from CWA to the persuasive system design framework, we took previous work [22,24] a step further by adopting the WWWWH paradigm to map the spectrum of the persuasion context to our CWA. This use of a WWWWH approach is similar to a previous approach by Mohr et al. [25] but establishes a link to a full ecological framework and toolkit from a well-known systems analysis framework. For each of the questions of the WWWWH paradigm, we linked appropriate sources of information from the CWA. We also captured *when* a change would occur by mapping information from the CWA to inform Fogg Behavior = Motivation + Ability + Trigger model [23]. Our WWWWH approach linked specific sources of information to describe the persuasive context, which could then be used by the persuasive system design to develop an effective design. The mapping of each framework to the WWWWH paradigm is shown in Table 1.

### Development of Persuasive Design

After defining the persuasion context, we identified several persuasive design principles that could help change the data-entry behavior [21]. These principles were selected from the persuasive system design framework [3] behavior. The persuasive design principles were incorporated into several different user interface designs and discussed with the organization. A final design was developed over the course of several months, during which drafts and comments were sent back and forth between our team and the organization until the design was acceptable to all parties. The design was then implemented and tested by the organization’s application developers.

### Evaluation of Impacts

The new design was published as an update to the organization’s reporting system. We measured the impact of the new design to measure the impact persuasive design had on user behaviors related to data entry.

**Table 1:** Linking cognitive work analysis to the persuasive system design’s persuasion context.

WWWWWH^a^ paradigm and Fogg Behavior Model		Persuasive system design framework			Analytical need	Cognitive work analysis context	Cognitive work analysis phase(s) and outputs
		Intent	Event	Strategy			
Who		Persuader	User	N/A^b^	Identify the persuader and the user or class of users who are the target of the persuasive intervention.	Ecological	Work domain boundaries (Phase 1) and social organization models (Phase 4)
What		Change type	Technology	N/A	Identify what behaviors need to change and what the new target action or behavior looks like.	Cognitive	Descriptive decision-making logic trees (Phase 2)
Why		N/A	Use	N/A	Contextualize the reasons for the task in the complex system. Why did old behaviors develop and what are the constraints on new behavior?	Ecological	Hierarchal relationships between ecological factors (Phase 1)
**When**							
	Motivators	N/A	N/A	N/A	Identify motivating factors within the ecology.	Ecological	Hierarchal relationships between ecological factors (Phase 1)
	Abilities	N/A	N/A	N/A	Identify user abilities and capabilities. Identify constrained resources (eg, time, money, etc).	Cognitive	Descriptive decision-making logic trees (Phase 2) and skill taxonomy (Phase 5)
	Triggers	N/A	N/A	N/A	Identify reasons users adopt specific behaviors or strategies.	Ecological	Descriptive decision-making logic trees (Phase 2) and strategies analysis (Phase 3)
How		N/A	N/A	Message route	How will we create a change? What design principles and strategies would be appropriate?	N/A	N/A

^a^WWWWWH: who, what, where, when, how.

^b^N/A: not applicable.

### Ethics

The study was submitted to the University of Waterloo Ethics Board and approved before the deployment of the new design and before the collection of data. To measure the impact of the new design, the study was positioned as a secondary analysis of data, as the organization was opting to independently deploy a suggested design. Data were collected by the organization, and users were not required to opt into the study because the change was implemented as part of the organization’s normal software revision and update cycle. We served a consultancy role to assess the impact of the change as a third party. All data shared for the study was deidentified, and we had no direct contact with users.

In order to collect direct feedback and comments, the organization identified contacts to discuss the results in semistructured interviews. These contacts completed consent forms. Users were also invited to complete an anonymous survey and consented to their participation.

Throughout the study, the new design did not endanger the availability of patient data or risk the organizations’ ability to report its activities. The design changes were considered passive and posed negligible risks to the organization, its users, and patients.

### Measures

To measure the impact of the summary screen on data entry, we calculated measures of record validity, completeness, and timeliness. These measures were developed collaboratively with the organization during a previous study of the same system [18]. Each of our measures is defined in Table 2.

### Statistical Analysis of Measures

To measure the effectiveness of the intervention, the field study was set up using a repeated measures experimental design. We analyzed data spanning a period of 16 weeks by looking at data before and after the new design was deployed. Each data quality measure was calculated for each user 8 weeks prior and 8 weeks after the implementation of the new summary screen. The measures for each user were averaged for the pre- and postperiods. The total number of entries (ie, patient visits) were compared between periods to ensure that the visit volume was not significantly different. Data from the premeasurement and postmeasurement periods were analyzed with a paired *t* test. *P* values, Cohen *d*, and power were calculated.

### Statistical Process Control

We expected noise within the dataset and assumed it could skew results positively or negatively. Noise in our measures, which were generated from a real-world, dynamic, sociotechnical system over the course of 16 weeks, would not be abnormal. For example, management meetings, programming changes, organizational behavior, strategic direction, and management priorities could easily change behaviors during the study. As well, it should be expected that patient volumes and care needs fluctuate seasonally and over the study period (eg, higher volumes for the flu in the winter and lower volumes for assessments around the holidays as staff use vacation time). We wanted to ensure that our statistical results were not attributed to normal changes or noise.

Measuring changes to variables within a “noisy” complex system is not a unique challenge in health care. This issue is often encountered when evaluating quality improvement initiatives in health care and is supported by the use of statistical process control (SPC) [26,27]. Thus, in our study, we contextualized the impact of our intervention by using SPC techniques to measure variance over time.

The notion of SPC is to measure process variance in 2 categories. The first type of variance in SPC is chance variation (also known as common cause variation). This category of variation is caused by phenomena that are always present within a system. Chance variation is anticipated noise associated with normal system operations. The second type of variance in SPC is assignable cause variation (also known as special cause variation). This category of variation is caused by phenomena that are not typically or historically present in a system. Assignable cause variation is associated with changes to the system’s operation [28].

A common display tool for SPC, the XmR chart, consists of 2 graphs. The first graph in the chart is a measure of a variable over time (X). This graph shows the mean calculated value for the analysis period, an upper control limit, and a lower control limit. A line graph is shown over a period of time. If values are above or below the control limits, they represent assignable cause variation. Values between the control limits represent chance variation. The second graph in the chart shows the moving range (mR) between each value in the X graph. A mean value for the period and a upper control limit are also shown. These graphs represent the absolute value of the change from period to period and can be used to identify significant variation. Variation above the upper control limit is abnormal [28].

**Table 2:** Data quality measures.

Measure name	Definition
Percent same-day entry	The percentage of entries that were entered on the same day as the appointment.
Percent complete	The percentage of entries that were measured as complete. An entry was considered complete if all fields had data and if the reason for the visit was not specified as “other.” If the visit was an initial encounter, the referral source was required.
Percent valid	The percentage of entries that were measured as valid. An entry was considered valid if the appointment date occurred before the entry date, if the appointment date occurred after January 1, 2008, and if the amount of time between the appointment date and entry date was <4 months. If the time between the referral date and the appointment date was greater than 6 months, it was considered invalid.

SPC is intended to be used when measuring change within a complex system with many sources of noise. Normally, SPC is used to measure quality improvement by a team and becomes part of an overarching quality improvement philosophy; teams use SPC during weekly or monthly meetings to track progress, identify potential signal changes and causes, and improve processes. To analyze behavior change in data entry, SPC lends itself well to putting any observed changes into context.

We created SPC XmR charts for each of our data quality measures. We generated XmR charts with the *R* statistical software and the qicharts package. The chart generation was scripted and automated to take data directly from a secondary Structured Query Language database that performed the data grouping. To give context to the results, the XmR charts were generated with data including 7 months before the intervention and used all available data following the intervention. All data points were used to calculate the average, upper control limit, and lower control limit values. The implementation of the user interface change was graphically marked on the XmR chart with a black line and the note “UI CHANGE.” Charts were created by breaking down the data by month.

### Feedback and Comments

After 8 weeks, users were invited to complete a survey. The survey included 3 free-text response questions, including Question A, “Do you have any comments about the reporting system?”; Question B, “Do you have any comments about the new summary screen?”; and Question C, “How could you be motivated to enter accurate, complete, and timely data into the reporting system? Did the summary screen help?”

Two managers who were familiar with the organization, its culture, and its initiatives were asked to comment on the patterns and changes visible in the XmR charts. Semistructured interviews and email correspondence took place after the design change had been deployed for 8 weeks.

## Results

### Persuasion Context

The results of our combination of CWA and the persuasive system design framework to define the persuasion context are shown in Table 3.

**Table 3:** Persuasion context.

Question		Answer	Referenced framework
Who		Our target users are health professionals entering data into the family health team reporting tool. There are no complex team dynamics as users enter data. The exercise is individual.	Described and modeled in the abstraction hierarchy phase of cognitive work analysis
What		At the alert level of the control task analysis, we want users to enter their data into the system after they have finished a patient encounter.	Described and modeled in the control task analysis phase of cognitive work analysis
Why		Summarizing the data is related to benchmarks and norms of the organization. The task will help the organization be accountable. Timely data will allow the organization to respond to needs more quickly. Professional values and training provide potential insightful constraints on the change. Building and moderating behavior through a sense of “duty” or by developing the sense of a professional norm could be a valuable approach to persuasive design.	Described and modeled in the abstraction hierarchy phase of cognitive work analysis
**When**			
	Motivation	Users have professional values which will lead them to input data. Users are responsible for meeting organizational benchmarks; failing to report data could result in disciplinary action.	Described and modeled in the abstraction hierarchy phase of cognitive work analysis
	Abilities	Users need to prioritize their time and engage in time management to change this behavior. They need time and time management abilities.	Described and modeled in the skill, rule, and knowledge taxonomy phase of cognitive work analysis
	Triggers	Users are triggered and influenced to record data by organizational policies, workload requirements, experience, technical abilities, and practice workflows.	Described and modeled in the strategies analysis phase of cognitive work analysis
**How**			
	Message	Persuasive messages should encourage users to enter data on the same day. The messages should appeal to each user’s sense of professional duty and desire to meet professional norms. Users need to be encouraged to think about entering data right away and avoid the bulk entry strategy. Users need to be encouraged to use the same-day workflow strategy.	Described and modeled in the strategies analysis phase of cognitive work analysis. Application of persuasion context analysis
	Route	The persuasive route can be direct or indirect.	Persuasion context analysis
Strategy		To reduce entry delay, a dialogue-based persuasion strategy could be appropriate. Effective approaches might include praise, rewards (computer-based), or suggestions. Reduce entry delay, a persuasion strategy based on social support, could also be appropriate. Effective design principles might include social comparison, normative influence, and social facilitation.	Persuasive system design framework

### Persuasive Design

#### Design Description

**Figure 2:**
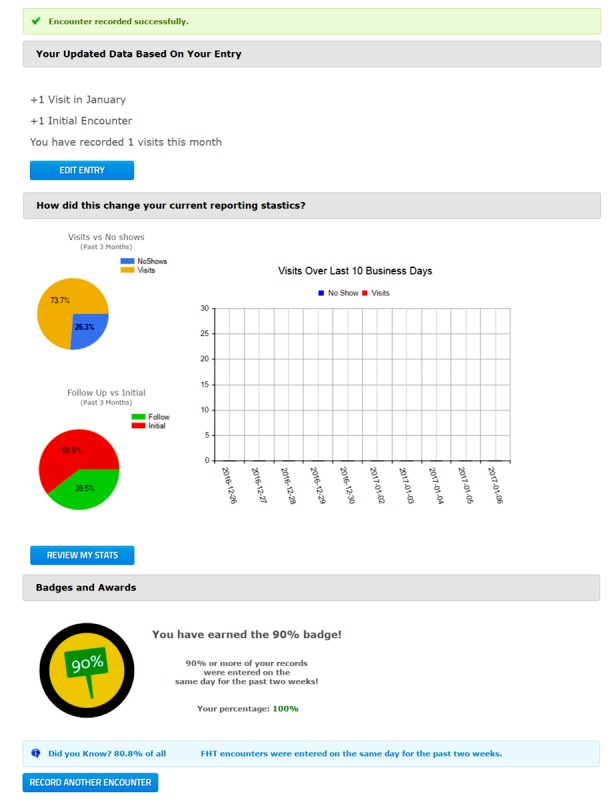
Screenshot of the persuasive summary screen.

The organization wanted an unobtrusive design that did not involve amending entry fields in the system’s input screen (see Figure 1). In the final design, the persuasive elements were introduced to the system through a new summary screen that was displayed after each user entered data. Whereas users normally clicked “RECORD ENCOUNTER” and were brought to a blank form, the change would now show a summary screen and ask users to click “RECORD ANOTHER ENCOUNTER” after reviewing the new content in the new design. The design of the summary screen was divided into 3 sections. A screenshot is shown in Figure 2.

#### Section 1: “Your Updated Data Based on Your Entry”

The first section of the screen is linked to the data validity and data completeness measures. This section supports data accuracy and completeness by inviting users to edit their submission, if anything is missing or incorrect, after showing a summary. An “Edit Entry” button was placed below the text to support the editing workflow; users can go back and make changes if an error was recorded or if something was missed.

This section was an adaptation of the verifiability and trustworthiness principles of the persuasive system design. In this context, users see what data they have inputted into the system and can see how it will be counted in reports. It aims to clarify how the data they inputted will be used.

#### Section 2: “How did This Change Your Current Reporting Statistics?”

Previous studies have found a positive relationship between use and data quality. For example, audit-based education proved to be an effective tool for improving data quality in primary care by providing users with a baseline during meetings, educating users about how data is used and recorded, and establishing goals [5]. Thus, increased attention and focus on data, engagement of stakeholders, and comparisons had positive impacts on data quality. Facilitating these processes would be a good use of persuasive design. In a previous study, there was a positive relationship between use and completeness for the reporting tool [18], suggesting that encouraging use would have positive impacts on data within the reporting tool.

This section aims to engage users with their data. As an alternative to users “using” their data through a report, the screen automatically shows important graphs, and in a sense, forces them to “use” their data. The design includes information that was deemed to be most important by the organization; a pie chart that broke down the user’s no-show rate from the last 3 months, the user’s follow-up ratio from the last 3 months, and a graph of scheduled visits (no-shows and actual encounters) over the last 2 weeks. Beneath these charts, users could click “Review My Stats” and generate more complex reports in the report module.

By engaging users with their data, the design aims to improve timeliness (ie, keep the data up to date for proper graphing) and encourages users to input valid and complete data to correctly display their data. The sentence “How did this change your current reporting statistics?” refers to how the user’s action and inputted data changes their statistics in reports. This was an implementation of the task support self-monitoring principle from the persuasive system design model.

#### Section 3: “Badges and Awards”

In the persuasive system design framework, the praise principle states that “by offering praise, a system can make users more open to persuasion” [3]. The section uses badges to encourage and normalize entering data on the same day. The persuasive design encourages users to think about keeping their statistics and encourages them to change their workflows and data-entry strategies accordingly.

The final iteration of the badges section shows a “same day” badge, which is programmed to display and reward the percentage of same-day entries. Different badges are presented with 70%, 80%, and 90% marks. The text provides a current same-day percentage measure. As long as a user remains between 90% and 100% same-day, they will keep the “top” badge available to them. This section also displays the percentage of users that enter their data on the same day. The message at the bottom is an implementation of the praise dialogue principle and the social facilitation principle from the persuasive system design model. This section aims specifically to encourage same-day entries.

### Impact Measures

#### Statistical Results

We collected data from all active users of the system (53 users), paired for the pre- and postperiods. We compared the number of entries completed in the 8 weeks prior to the change and the 8 weeks after the change. The average number of entries per user for the preperiod was 336.62 and for the postperiod was 314.31. The difference of 22.31 entries was not significant (*P*=.23). Thus, the pre- and postperiods were similar in terms of the volume of patient visits and data collection. The results of the paired *t* test of each data quality measure are presented in Table 4.

According to the pre-post analysis, the intervention increased the percentage of same-day entries by 10.3%. The test was statistically significant (*P*<.001) with a power of 0.999. The Cohen *d* of 0.70 would be considered a large effect.

According to the pre-post analysis, the intervention decreased the percentage of complete records by 4.8%. The test was statistically significant (*P*<.001) with a power of 0.957. The Cohen *d* of 0.505 would be considered a large effect.

According to the pre-post analysis, the intervention increased the validity measure by 0.7%. The test was (marginally) statistically significant (*P*=.05) with a power of 0.537.

**Table 4:** Pre versus post results with paired *t* tests.

Records	Pre (%)	Post (%)	Change (%)	*P* value	Power	Cohen *d*
Same-day entries	62.8	73.2	+10.3	<.001	0.996	0.632
Complete records	86.3	81.6	−4.8	<.001	0.978	0.545
Validity measure	98.9	99.6	+0.7	.05	0.537	0.282

#### Control Charts

To understand the changes to the data measures in the context of noise, we created XmR charts for same-day percentage, completed percentage, and validity percentage. Data for the XmR charts were grouped into months. Data from 7 months prior to the change and 3 months afterward were included to contextualize historical system noise and put the changes after the design change into a larger context. The user interface change took place in the last week of November 2016.

**Figure 3:**
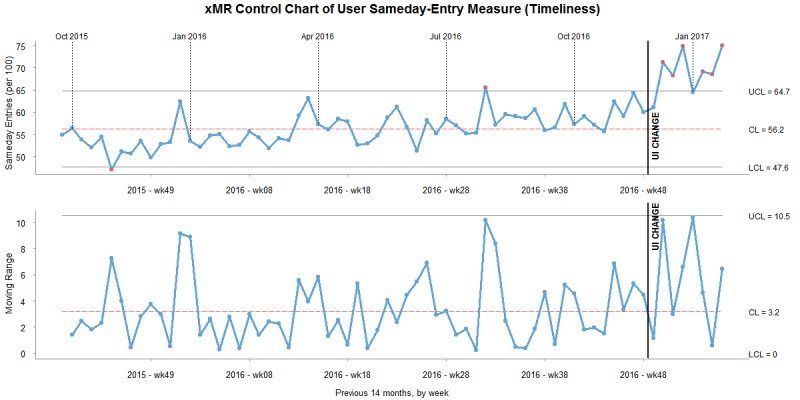
XmR chart of timeliness measure. CL: control limit; UI: user interface; UCL: upper control limit.

As shown in Figure 3, the same-day percentage average rose above the upper control limit after the change. For 3 months after the change, the same-day percentage were almost all above the upper control limit, which can be attributed to assignable cause variation and was not associated with normal variation or “noise” in the system. The mR (eg, the change from month to month) was high immediately after the change. The perceivable increase in the timeliness measure is consistent with the statistical results.

**Figure 4:**
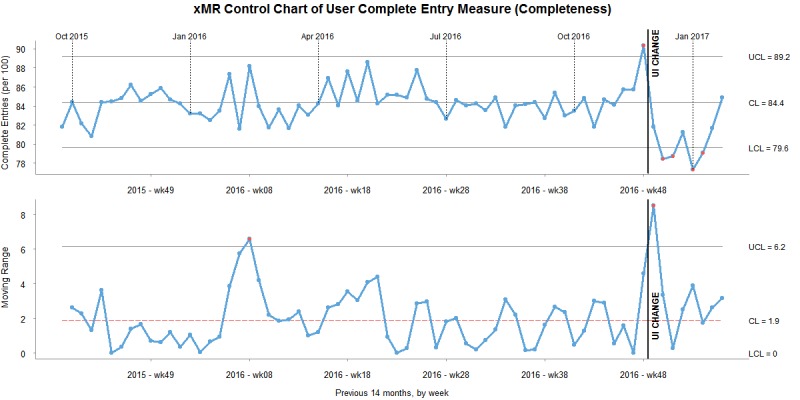
XmR chart of completeness measure. CL: control limit; UI: user interface; UCL: upper control limit.

As shown in Figure 4, the complete percentage did not rise above the upper control limit after the change. In fact, the values dropped below the lower control limit. As the values dropped below the control limits, the changes represent assignable cause variation and were likely caused by the change. By the third month after the change, the completeness measure returned to a midrange point. The mR (eg, the change from month to month) was very high immediately after the change. This indicates that the change to the user interface impacted and changed the measure in a significant way. The perceivable drop in the completeness measure is consistent with the statistical results.

**Figure 5:**
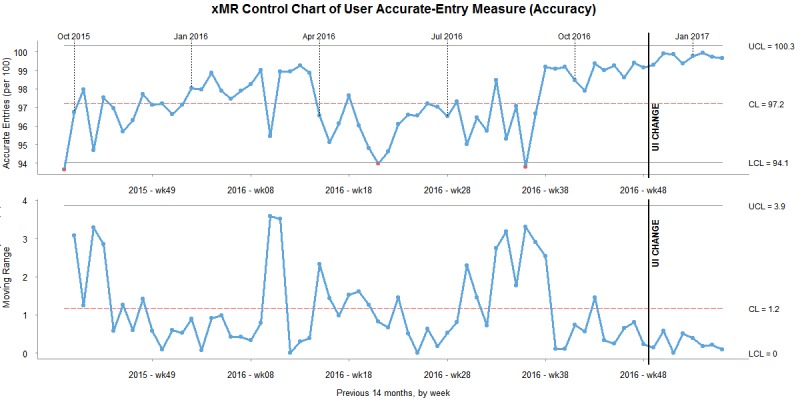
XmR chart of validity measure. CL: control limit; UI: user interface; UCL: upper control limit.

As shown in Figure 5, the validity percentage average rose above the upper control limit after the change. For 3 months after the change, the validity percentage stayed within the control limits and could be associated with normal variation or “noise” in the system. The mR did not rise above the upper control limit or below the lower control limit. Based on this chart, the change did not impact the data’s validity. The changes in the validity measure were consistent with the statistical results, which were marginal with a *P* value of 0.5.

### Feedback and Comments

#### User Feedback

A total of 17 users completed the survey that was distributed, and 13 of those users provided comments to Question B, “Do you have any comments about the new summary screen?” and Question C, “How could you be motivated to enter accurate, complete and timely data into the reporting system? Did the summary screen help?” Relevant responses to each question are shown in Table 5 and Table 6. A complete set of responses are available in a published dissertation [21].

**Table 5:** Responses to Question B.

Respondent	Comment
5	It makes me feel anxious and unhappy to see a lot of no shows.
6	The new screen data seems to put more unnecessary pressure on data entry.
8	Even though it only takes a few seconds for the new screen to load and then a few more seconds to click “record encounters” and for that screen to load, it really adds up! [Entering data] seems to take way longer now.
9	I like seeing the graphs — I'm a visual person, and this helps to summarize what I view as important info about my practice.
10	Please remove — adds time to data entry and doesn't change practice.
11	I would prefer to see the summary screen once only when I start to enter data [...]
14	Seems unnecessary.
15	I don't need to see my percentages page after entering each client encounter. Could be used as a summary page of day/week/month. Easy to read and understand.
16	The summary needs only to come up when I have completed all entries, not after every [patient] encounter [because it] takes too much time.
17	The new summary screen added lag time to inputting stats, and [has made] the process [more] cumbersome.

**Table 6:** Responses to Question C.

Respondent	Comment
5	I always have entered my data on the same day. Summary screen just makes me anxious.
6	I have usually recorded data on the same day. The new screens seem to discourage that.
7	I personally did not see any difference.
8	It helped slightly. I find I am now entering stats every 7-9 days instead of every 9-14 days.
9	I'm not sure it provided extra motivation; I'm a pretty organized person so have always wanted to keep on top of doing stats.
10	Monetary rewards [would be motivating]. [...] The summary screen did not help [motivate me].
11	At first [the new screen] helped somewhat; now I again rely on my own motivation to keep up to date, which ebbs and flows with the demands of my schedule.
13	[The new screen helped] a bit.
15	I was already entering data on daily basis, but I do feel it could act as a motivator to those who have not in recent past.
16	I know I need to enter my work into [the reporting tool] but I am not particularly motivated to do so, not sure what would motivate me.
17	It was nice to see incentives on the screen of reaching goals and receiving badges, but other incentives would likely help motivate.

#### Comments by Management

The 2 managers designated as contacts were asked to comment on the drop in the completeness measure. The Information Systems Manager responded as follows:

It appears it is because of the referral issue.

There is an option where you can [click] ‘I do not know when the referral source was’ [and the system, therefore, records] the date of 1900-01-01.

[We implemented this feature because] staff pushed back saying that they do not always have the referral date handy so they need [the ‘I do not know’] option...

If [users] leave the default option of ‘I know when the referral date is ‘[the system] forces [users] to [enter] a date’. Based on the data, it seems that more users have started to click the ‘I do not know the referral date’ option, which seems to explain the change in the completeness measure.

Information Systems Manager, personal communication, February 16, 2017

The managers were also asked to comment as to whether or not other interventions, meetings, or policies around data entry had changed over the course of the study period. They noted that there was no formal or direct intervention around data entry in the reporting system other than the user interface change.

## Discussion

### Objective

Our objective was to find a way to influence clinician behaviors around data entry and to improve data quality. Our approach was to expose users to a new design that adopted persuasive design principles in order to modify their data-entry behaviors. Based on our results, there is evidence that a behavior change took place for users of the reporting system as a result of the design change.

### Impact of Design Change

#### Data Quality Measures

There were changes immediately after the new design was deployed in each of the 3 data quality measures used to measure the impact of the design change. Though these measures were not perfect representations of timeliness, completeness, and validity, they were suitable proxies. In a previous study, the improvement of these measures represented measurable enhancements to data quality [18].

Obviously, there may be questions about whether the observed changes can be attributed to the changes to the user interface. The organization had an interest in improving data quality, and various meetings, staff instructions, or other events over the study period could have contributed to the improvement. We attempted to contextualize this potential problem by using SPC control charts and by discussing this potential problem with the management contacts.

The SPC control charts are intended to help differentiate changes related to noise and “normal” changes within the system. We attribute any meetings, staff instructions, or other events related to data quality as “normal” system noise. The SPC chart contextualizes normal changes when values occur between the control limits. Based on the data available for the previous year, any events that could have changed data quality fell within these bounds. The user interface change was a unique event and pushed the measures above the upper control limits, meaning that something occurred outside the normal system “noise” and that the values were significant and could be assigned a cause. Since management commented that there were no other events during the study period that could have influenced the measures, there is good evidence that the user interface design change very likely impacted the measures. These conclusions are supported by the *t* tests and significant statistical inferences.

The impacts of the design change did not work exactly as intended. The timeliness measure improved, the completeness measure worsened, and the validity measure showed a marginal (if any) change.

#### Timeliness Measure

The XmR chart for the timeliness measure tells a compelling story. Before the change, the system signal was relatively stable. Small spikes occurred before the end of each quarter, which managers associated with peak reporting periods and seasonal organizational pressures. Before the implementation, there were no other obvious trends and no out-of-control signals. After the change, all values were above the upper control limit. There was a significant change after the user interface change, based on the mR graph. Based on the results of the XmR charts and the paired *t* test, the evidence is compelling that the intervention increased the number of same-day entries within the system.

#### Completeness Measure

The XmR control chart for the completeness measures shows that the completeness measure was a relatively stable measure over the previous 7 months. There were no noticeable spikes or changes, and no trends or out-of-control signals were seen. The month before the implementation, the completeness measure hit a high point. A significant impact on the completeness measure can be seen when the intervention was deployed. The impact was significant, as shown in the mR graph. Interestingly, it appears that after the initial “shock” of the change, the completeness variable appears to be returning to normal. The results of the *t* test and XmR are consistent.

Based on the comments from the organization’s managers, it appears that immediately after the summary screen introduction, the completeness measure was reduced because there was a significant change in a number of entries recorded with the “I don’t know the date” instead of entering the referral date for initial encounters. It is very interesting that a passive change to the user interface (a noninteractive summary screen) changed user behavior in this way. The summary screen appears *after* users enter the information and select referral details. It appears that a statistically significant number of users responded independently to the intervention in the same manner. This may represent a reaction to the ”same day” badge on the summary screen: to hit this metric as quickly and easily as possible, users abandon the referral date to optimize their time. This tradeoff is consistent with behaviors modeled in the CWA [21]. To counterbalance this adaptation, a ”completeness” badge may be appropriate.

#### Validity Measure

Interestingly, the results of the validity values are comparable to the results from other medical registry case studies, which have reported 98% accuracy (ie, validity) based on a gold standard [29]. The data show that there was a significant change in the percentage of valid records.

It is important to put these improvements in context, as they were relatively small. The paired *t* test did not have a strong statistically significant result compared with the other measures. The Cohen *d* of 0.282 would be considered a small-to-medium effect. Though the validity measure in the XmR chart shows an improvement, rejecting the null hypothesis and concluding that there was a significant impact should be cautiously done.

### User Comments

Several users articulated positive feedback and gave the intended behavior change heuristics that the summary screen was intended to encourage. For example, “I find I am now entering stats every 7-9 days instead of every 9-14 days,” “I like seeing the graphs — I'm a visual person, and this helps to summarize what I view as important info about my practice,” “It was nice to see incentives on the screen of reaching goals and receiving badges,” and “I feel it could act as a motivator to those who have not [been timely] in the past.” Some users suggested there was only an initial impact with comments such as “At first it helped somewhat; now I again rely on my own motivation.” These comments align with the persuasive system design proposed by Oinas-Kukkonen and Harjumaa [3]: there are different kinds of behavior change (eg, one time, short-term, long-term), and different kinds of interventions are appropriate for each. While it is clear that the summary screen introduced a change in behavior and influenced the users, further work will be required to properly categorize the change as either short-term or long-term.

Other comments in the survey were concerning. One user reported that it felt like there was new pressure on data entry. This is not an incorrect impression, but associating pressure to enter data with the summary screen was unexpected. It appears some users saw the summary screen as an accentuation of historic management reminders to enter data on time and had a negative reaction. This is further described by another respondent who said the summary screen made them feel “anxious and unhappy” and complained that “the summary screen just makes me anxious.”

Anxiety and unhappiness from users are very strong words. However, the true cause of anxiety is not the summary screen or the data, but the user’s performance and statistics. Specifically, the user complained that the summary screen caused anxiety because the system reminded them that they had no-show visits on their record. This would be akin to a student expressing anxiety over seeing their grades posted on a learning management platform. Regardless, if users feel that the summary screen is tracking their progress closely as a proxy manager, it is understandable that performance tracking could cause anxiety.

Contrasting responses were provided regarding the summary screen. Whereas some users expressed seeing a carrot, others saw a stick. Based on the data and outcomes, this would be an example where performance and preference are not correlated; it appears performance is occurring where preference is not.

### Design Improvements

The final design used in the study was developed in collaboration with the organization. As it is in many cases and was also in this case, certain compromises were made, and the organization was the best expert on what kind of solutions should be provided for their employees and how to make changes without causing any problems. Having said this, there are several possible iterations for the design.

Five comments mentioned concerns about the performance impacts of the summary screen, including “adds time and doesn’t change practice”, “The summary screen added lag time and [made the process] more cumbersome,” “[The extra time required] really adds up!”, and “It feels slower to load pages and enter data.” Based on these comments, there does appear to be a concern about the performance of the system. The organization has since taken this feedback and adjusted their queries with table-valued functions to reduce the load time by 80% (Information Systems Manager, personal communication, February 16, 2017).

In other comments, users provided suggestions for user experience and user interface adjustments to the summary screen. For example, 2 users suggested having the summary screen appear only once a day, instead of after every encounter, or enabling a daily, weekly, and monthly view. These suggestions are not unreasonable and could be implemented by the organization in a software revision. Taking the summary screen out of the workflow would address most of these concerns, but it is not clear if this would continue to provide the same effect on user behavior.

In terms of improving the summary screen, a few design heuristics may help alleviate some of this anxiety. Currently, the data provided is only a measure of a single user’s data. Comparisons between groups and users might help alleviate performance anxiety by normalizing their results. If a user is worried about their performance, would it not be helpful for them to see the performance of other similar users? A comparison paradigm could help build a user’s confidence, compliance, and engagement and reduce potential anxieties about their own data. Further work and study would be required, however, as there is also the possibility that comparisons could increase anxiety by making users defensive about their performance and feel inadequate about their statistics compared to peers. The comparison concept is part of the persuasive system design model, which describes normative influence and social facilitation as design principles. This idea would not be difficult to incorporate into the new summary screen and design change.

### Contributions

There are many studies in the literature demonstrating that persuasive design can be useful for changing patient attitudes and behaviors through mobile devices. However, there are few examples of persuading clinical users to change their behavior. Knowing that primary care data recording behaviors impact data quality and its secondary uses and that these behaviors are impacted by the enthusiasm of clinicians [17], our study demonstrates a novel path forward. Our study is a unique contribution, demonstrating that persuasive design techniques are viable tools for changing not only patient behaviors in the health care system but also clinicians and system users.

Knowing that social, intraclinician comparisons have been effective approaches for changing clinician data-entry practices [5-7], our work shows the viability of using persuasive design to emulate those types of social mechanisms with technology. In the future, persuasive design could be used to encourage adoption and use and manage the organizational and social aspects of successful clinical system implementations. The use of persuasive design with clinicians is an exciting and interesting area for further study. It will be very interesting to vendors and developers in the health care ecosystem.

Importantly, this work makes a major contribution by describing *how* persuasive design can be used in design to achieve a specific goal. This was done by combining the persuasive system design model and CWA with a WWWWH paradigm and extending previous work [22,24]. Our research showed that building a persuasion context using a detailed systems analysis framework can facilitate the deployment of effective interventions and that these interventions can influence behaviors in users in intentional ways.

### Limitations

Our study is unique because it involves a combination of theoretical work and a real-world “in the wild” evaluation of the design. This combination introduced constraints to the study, which could be improved and extended in several ways.

One area that would have been interesting to explore is variation between users and groups of users. Unfortunately, we were limited to 53 users in our study. Because these users worked in multiple locations and could be categorized into 1 of 8 different professions, any comparisons would rely on very small groups and would not permit meaningful, statistically significant comparisons. In the future, if the reporting system were deployed into additional organizations, these types of comparisons could be both possible and quite interesting.

The length of the postperiod is limited to 8 weeks for the *t* test comparisons and 3 months in the XmR charts. The issue of short-term versus long-term impacts on behaviors is obviously of interest to the academic community [3], and an evaluation of long-term impacts of the design change and comparisons to short-term impacts would be valuable. Unfortunately, the scope and funding of the study did not allow for a longer-term evaluation of the metrics and the long-term impacts of the design change. Future work will involve exploring and evaluating the long-term impacts in greater detail as well as assessing iterative improvements to the design.

### Conclusions

The reporting tool used by allied health professionals in a family health team provided us with an interesting opportunity to explore the use of persuasive design to change clinician attitudes and behaviors regarding data entry. We demonstrated that informing persuasive design with CWA can be effective in designing an intervention that can change data-entry behavior and reduce entry delay. Our study demonstrates merit to the use of persuasive design for changing data-entry behavior in clinicians. Further work is required to perfect and test additional designs. Persuasive design is a viable approach for designing and encouraging behavior change and could support effective data capture in the field of medical informatics.

## Abbreviations

CWA: cognitive work analysis
mR: moving range
SPC: statistical process control
WWWWH: who, what, when, where, how
